# Immunological fortification at our barrier organs: Protecting us as we age

**DOI:** 10.1111/imm.13197

**Published:** 2020-05-13

**Authors:** James A. Harker, Laura J. Pallett

**Affiliations:** ^1^ Faculty of Medicine National Heart and Lung Institute Imperial College London London UK; ^2^ Division of Infection and Immunity Institute of Immunity and Transplantation University College London London UK

## Abstract

Our barrier surfaces are fundamental in protecting us from the outside world and segregating key biological processes. The immunological fortifications found at these sites therefore possess many distinct qualities, which are discussed in *Immunology*'s series of reviews on Barrier Immunity. Together these reviews showcase novel biological processes identified through the use of state‐of‐the‐art technologies, and specifically highlight how these change throughout our lives.

The barriers of the human body represent our first line of defence against the outside world. Our barrier organs therefore face the formidable immunological challenge of defending us against pathogenic insults and promoting a peaceful co‐existence with the local microbiota that inhabit them. Most antigens faced by our immune system initially enter through breaches in the physical barriers of the skin, or across one of the mucosal surfaces of either the respiratory, gastrointestinal or urogenital tracts. Consequently, these tissues are ‘immunologically fortified’ with common and distinct features, and their preservation is an essential component of host survival.

This is beautifully illustrated in the female reproductive tract (FRT). In their review of immune responses in the FRT, Monin *et al.* ^1^ highlight its dual identities. The lower regions of the FRT including the vagina are characterized by a multi‐layered stratified squamous epithelium, a mucus layer containing antimicrobial peptides, antibodies and immune mediators, and a substantial *Lactobacillus‐*rich microbial community, forming a substantial physical and chemical barrier against invasive pathogens. This is complemented by a full arsenal of immune cells, including substantial populations of tissue‐resident memory T‐cells*. *The upper parts of the FRT, the uterus and ectocervix, meanwhile contain only a single layer of epithelium and appear devoid of commensals. Instead, the uterus possesses an immune system that is adapted to balance mucosal immunity against microbial exposure and immune tolerance, allowing and promoting the growth of a fetus. Indeed recent use of single‐cell RNA sequencing (scRNA‐seq) has revealed distinct populations of decidual natural killer (NK) cells^2^ which, as Male and colleagues discuss, unlike their cytotoxic contemporaries in the circulation are poor killers but produce pro‐angiogenic and trophoblast chemoattractant factors, key to fetal development, confirming the importance of barrier immunity from the moment of conception.

With this in mind, Bottling and Haniffa reveal how the most overt physical barrier, the skin, begins to develop *in utero *(REF – Bottling *et al*.). Harnessing recent advances in scRNA‐seq with knowledge generated through traditional approaches such as histology allows the authors to take us on a molecular and cellular exploration of the human skin from the earliest points of life.^3^, ^4^ They discuss how the immune response of the skin *in utero* is drastically distinct from that seen after birth, and rapidly changing between each trimester. The review shows that while fetal skin is hypo‐responsive to inflammatory stimuli, it is primed to drive remodelling and repair; containing populations of type‐2‐like macrophages and dendritic cells, ILC2s and regulatory T‐cells (Tregs) that all promote wound healing without scarring in the 1st and 2nd trimesters.

The UK’s Office for National Statistics estimates that over the next 50 years an additional 8·2 million people will be living over the age of 65 years – that’s an increase the size of current‐day London. In their review, Chambers and Vukmanovic‐Stejic take an elegantly simplistic approach discussing the individual components of the stromal, innate and adaptive effector cells compartmentalized within the three layers of the skin in the steady‐state, and how these change as we age.^5^ Of interest to many of us in an ever‐aging society, the authors describe specific age‐related ‘defects’ specific to the skin barrier. For example, with increasing lifespan comes a host of complications resulting from chronic low‐grade inflammation termed ‘inflamm‐aging’. Alongside a loss of the structural integrity of the skin itself, a thinning of the epidermis and fragmentation of the extracellular matrix occurs, driven by increased matrix metalloproteinases and a reduced production of pro‐collagen. In addition, aging results in a significant reduction in local Langerhans cells and functionality of the local antigen‐specific T‐cell population, resulting in a greater incidence of bacterial or viral infections and cancer.

The immunological defences of the human skin provide protection to a 1·5–2‐m^2^ area. In comparison, the respiratory tract covers an area of 70 m^2^ that for the most part is made up of a single layer of epithelial cells for gas exchange. Maintaining efficient uptake of O_2_ and removal of CO_2_ means limiting the potential of immune cells to infiltrate this epithelium, even in response to infection. Invernizzi, Lloyd and Molyneaux focus their review on the ability of the respiratory epithelium itself to regulate immunity (REF – Invernizzi *et al*.^6^). Invernizzi *et al.* also highlight how recent 16S sequencing data of the lower airways has changed scientific dogma: what was once believed to be a largely sterile microenvironment is now considered an ideal niche for specific species of commensal microbiota. They also discuss how disruption of microbial homeostasis at the respiratory epithelium drives the pathogenesis of a number of lung diseases ranging from asthma to idiopathic pulmonary fibrosis.^7^ The lungs also play host to unique resident immune cells, most notably alveolar macrophages that populate the luminal side of the alveoli and airways. So distinctive are alveolar macrophages that they cannot currently be generated *in vitro*, and Willinger and colleagues use their review to examine emerging evidence from model systems including the use of humanized mice engineered to express human macrophage colony‐stimulating factor (M‐CSF) and granulocyte‐macrophage colony‐stimulating factor (GM‐CSF) to highlight additional developmental cues vital to their generation *in vivo*.^8^, ^9^ Further to this, the authors also discuss a number of dynamic changes that occur to lung‐specific macrophages occupying different niches throughout life, that start out as hypo‐responsive sentinels in early life, but can become hyper‐reactive as we age – potentially contributing to age‐related inflammatory diseases.

From an immunological perspective, perhaps the most extensively researched, discussed and reviewed barrier site has been the gastro‐intestinal (GI) tract. The GI tract is the barrier through which the majority of our nutrients flow, and is home to the canonical microbiome, containing over 10^12^ microbiota containing a wide range of parasitic, bacterial and viral pathogens (recently the focus of another review series^10^). In their review on integrin‐mediated activation of transforming growth factor (TGF)β, Travis and colleagues highlight the importance of the α_v_ integrin family in providing contextual signals, facilitating these diverse activities.^11^ They highlight the distinct roles played by the integrin‐TGFβ pathway at discreet barriers, driving tolerance, and limiting T‐cell responses. Perhaps of particular interest in the current setting of COIVD‐19, the authors also discuss the implications of over‐active TGFβ in limiting antiviral immunity and promoting specific disease pathogenesis within the lung.

Finally, our largest internal organ, the liver, is discussed by authors Swadling and Stamataki. The liver co‐ordinates many physiological processes, including the filtration of blood, metabolism and storage of macronutrients, and detoxification. Blood flowing to the liver transits via the GI tract and is therefore rich in antigens. As such, the liver is tolerized to avoid immune responses against innocuous antigens (similar to the upper FRT), whilst maintaining the ability to elicit immune responses to blood‐borne pathogenic insult.^12^ The authors discuss how emerging evidence from state‐of‐the‐art single‐cell technologies contributes to our understanding of this barrier (REF – Swadling *et al*.). Until recently, accurate mapping of the immune compartment in the liver was considered challenging as it required access to difficult/precious samples and high‐level experimental resolution. Describing the latest tools used to examine liver immunity in unprecedented detail, the diversity in the function and phenotype of resident immune cells, and heterogeneity of the parenchyma, is explained. Notably, the authors draw parallels between the murine and human liver, revealing how single‐cell analyses have advanced or redefined our understanding of immune responses at this barrier.

As so starkly highlighted by the on‐going global SARS‐Cov2 pandemic, we rely on the dynamic immunological homeostasis that occurs at our immunological fortified barriers every day. This collection of reviews highlight the use of novel technologies (microbial sequencing, metabolomics and single‐cell transcriptomics) and model systems to reveal the complex tapestry of cellular and molecular interactions occurring at each of our barriers in health and disease. With the advent of collaborative, open‐source initiatives such as the Human Cell Atlas,^13^ the next decade promises to be an exciting era in our understanding of how local immune cell ‘experts’ contribute to barrier immunity in both health and disease.
